# Extracellular vimentin interacts with insulin-like growth factor 1 receptor to promote axonal growth

**DOI:** 10.1038/srep12055

**Published:** 2015-07-14

**Authors:** Michiko Shigyo, Tomoharu Kuboyama, Yusuke Sawai, Masahito Tada-Umezaki, Chihiro Tohda

**Affiliations:** 1 Division of Neuromedical Science, Department of Bioscience, Institute of Natural Medicine, University of Toyama, 2630 Sugitani, Toyama 930-0194, Japan; 2 Division of Chemo-Bioinformatics, Department of Translational Research, Institute of Natural Medicine, University of Toyama, 2630 Sugitani, Toyama 930-0194, Japan

## Abstract

Vimentin, an intermediate filament protein, is generally recognised as an intracellular protein. Previously, we reported that vimentin was secreted from astrocytes and promoted axonal growth. The effect of extracellular vimentin in neurons was a new finding, but its signalling pathway was unknown. In this study, we aimed to determine the signalling mechanism of extracellular vimentin that facilitates axonal growth. We first identified insulin-like growth factor 1 receptor (IGF1R) as a receptor that is highly phosphorylated by vimentin stimulation. IGF1R blockades diminished vimentin- or IGF1-induced axonal growth in cultured cortical neurons. IGF1, IGF2 and insulin were not detected in the neuron culture medium after vimentin treatment. The combined drug affinity responsive target stability method and western blotting analysis showed that vimentin and IGF1 interacted with IGF1R directly. In addition, immunoprecipitation and western blotting analyses confirmed that recombinant IGF1R bound to vimentin. The results of a molecular dynamics simulation revealed that C-terminal residues (residue number 330-407) in vimentin are the most appropriate binding sites with IGF1R. Thus, extracellular vimentin may be a novel ligand of IGF1R that promotes axonal growth in a similar manner to IGF1. Our results provide novel findings regarding the role of extracellular vimentin and IGF1R in axonal growth.

Vimentin, a cytoskeletal protein belonging to the family of intermediate filament proteins, is generally known as an intracellular protein involved in cell adhesion and cell migration^1^,^2^. However, reports have shown vimentin secretion from astrocytes or macrophages. Mor-Vaknin *et al.*^3^ reported that vimentin is secreted from activated macrophages and is involved in a bactericidal effect. In a cortical astrocyte culture, vimentin was detected in astrocyte-conditioned medium (ACM)^4^. In another report, ACMs from the cultures of rat cortex, hippocampus or midbrain were administered to neural precursor cells, and neuronal differentiation was enhanced by the ACM from the culture of each region. In the ACM, vimentin was detected as an astrocyte-secreted protein in all three brain regions^5^. However, a specific role for vimentin secreted from astrocytes had not been identified. We recently demonstrated that vimentin was secreted from astrocytes and induced axonal growth^6^. However, the mechanism of the extracellular vimentin-induced axonal growth remains unclear. Therefore, investigating the signalling mechanism of extracellular vimentin may elucidate a novel type of signalling pathway for axonal growth.

In the present study, we aimed to clarify the mechanism of extracellular vimentin-induced axonal growth and investigated which molecules participated in this process. Our results demonstrate that extracellular vimentin binds to insulin-like growth factor 1 receptor (IGF1R), leading to axonal growth. In addition, IGF1R activation by vimentin is a direct action and does not occur via the secretion of known IGF1R ligands, such as IGF1, IGF2 or insulin. Thus, extracellular vimentin may be a novel ligand for IGF1R. This is the first report of a receptor molecule mediating the effect of extracellular vimentin in neurons. Interestingly, IGF1R can bind different types of ligands from the insulin family, as evaluated by extracellular vimentin. Our results suggest that extracellular vimentin-IGF1R signalling is a new pathway for axonal growth.

## Results

### Effect of vimentin on axonal growth was diminished by the blockade of IGF1R in primary cultured cortical neurons

Our previous study showed that vimentin was an axonal growth facilitator, however, the underlying mechanism was unknown. In many signalling pathways, some proteins are phosphorylated after stimulation with extracellular factors. To explore the molecules related to the extracellular vimentin-elicited signals, we investigated phosphorylated proteins in vimentin-treated cultured neurons using a comprehensive antibody array. Rat primary cultured cortical neurons at 4 days *in vitro* were treated with vimentin (100 ng/ml) or vehicle solution (control) for 10 min, and the cells were lysed with extraction buffer. The cell lysates were reacted with phospho-specific antibodies on array slides as described in the Materials and Methods section. The levels of the phosphorylated form of each protein were adjusted to the total protein expression levels (phosphorylated plus non-phosphorylated protein). The phosphorylation levels were compared between the control and vimentin-treated groups, and many proteins were phosphorylated by vimentin treatment. We hypothesised that extracellular vimentin conveys signals via certain receptors; therefore, we focused on receptor proteins among the phosphorylated proteins. Of these proteins, IGF1R exhibited the highest increase in phosphorylation, at 147% compared with the control. Therefore, we focused on IGF1R as a candidate of vimentin receptor.

**Figure 1:**
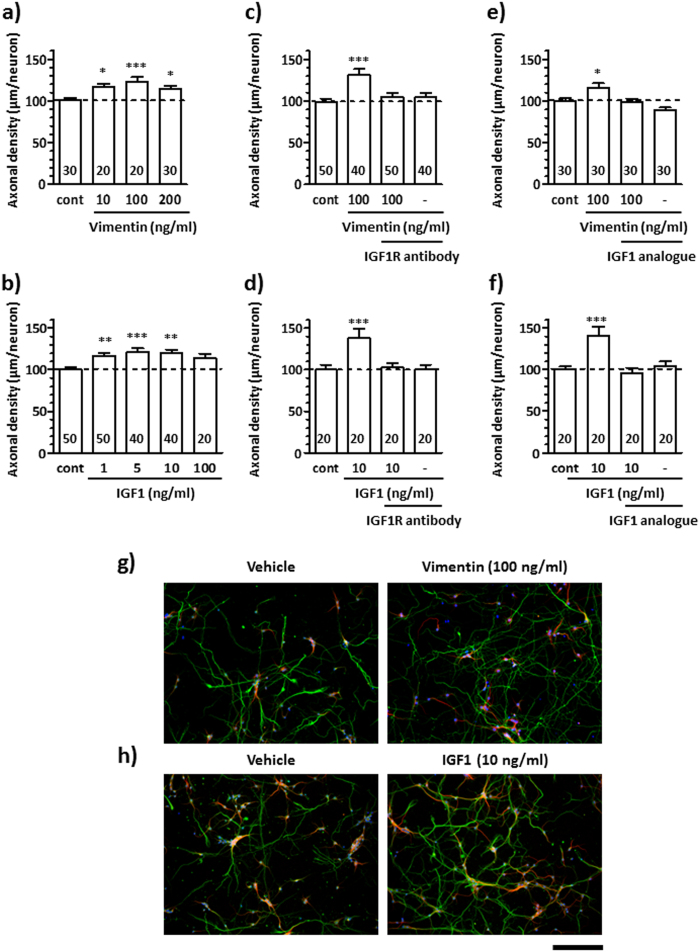
IGF1R-neutralising antibody or IGF1 analogue diminished vimentin- or IGF1-induced axonal growth. Cortical neurons were cultured for 1 day *in vitro* (div) and then treated with vimentin (**a**) at 10, 100, 200 ng/ml and IGF1 (**b**) at 1, 5, 10, 100 ng/ml. After pre-treatment with an IGF1R-neutralising antibody (2 μg/ml) (**c,d**) or IGF1 analogue (20 μg/ml) (**e,f**) for 15 min at 1 div, vimentin (100 ng/ml) (**c,e,g**) or IGF1 (10 ng/ml) (**d,f,h**) was administered to the cells. Six days after treatment, the cells were fixed and double-immunostained for pNF-H (green) and MAP2 (red) and counterstained with DAPI (glue). The density of pNF-H-positive axons per MAP2-positive neuron was quantified in each treatment. ^*^p < 0.05, ^***^p < 0.001, one-way ANOVA *post hoc* Dunnett’s test; numbers in columns indicate the number of photos for measurements. Representative images of pNF-H-positive axons in (**a**) and (**b**) are shown in (**g**) and (**h**) respectively. The scale bar indicates 200 μm.

To confirm the involvement of IGF1R in vimentin signalling, we investigated the effects of IGF1R inhibitors on axonal growth. Vimentin and IGF1 increased axonal densities in a dose-dependent manner (Fig. 1a,b). The results showed that 100 ng/ml vimentin and 10 ng/ml IGF1 treatment significantly increased axonal density. These concentrations of vimentin and IGF1 corresponded to 1.75 nM and 1.31 nM, respectively. Hereafter, we used vimentin and IGF1 at the doses of 1.75 nM (100 ng/ml) and 1.3 nM (10 ng/ml), respectively. An IGF1R-neutralising antibody^7^ (Fig. 1c,d) or IGF1 analogue^8^ (Fig. 1e,f) was used to inhibit IGF1R function. Vimentin (Fig. 1c,e) or IGF1 (Fig. 1d,f) significantly increased axonal densities in the absence of these inhibitors. In contrast, the vimentin-induced and IGF1-induced axonal growths were diminished by pre-treatment with an IGF1R-neutralising antibody (Fig. 1c,d) and IGF1 analogue (Fig. 1e,f). Representative images of axons in vehicle-, vimentin- or IGF1-treated group (Fig. 1a,b) are shown in Fig. 1g,h, respectively. The cell viabilities when axonal growths were measured in Fig. 1a to f were also evaluated (Supplemental Figure S1). No significant differences were observed in neuron numbers among groups either in response to vimentin or IGF1 treatments. The neuronal cell numbers were not changed by each treatment with various concentrations of vimentin or IGF1 for 24 h. (Supplement Figure S2). These results suggest that the axonal growth effects of vimentin and IGF1 are not related to the promotion of neuronal survival. Thus, our findings suggest that IGF1R is involved in vimentin-induced axonal growth.

### IGF1R phosphorylation peaked at 30 min after vimentin treatment in cultured cortical neurons

**Figure 2:**
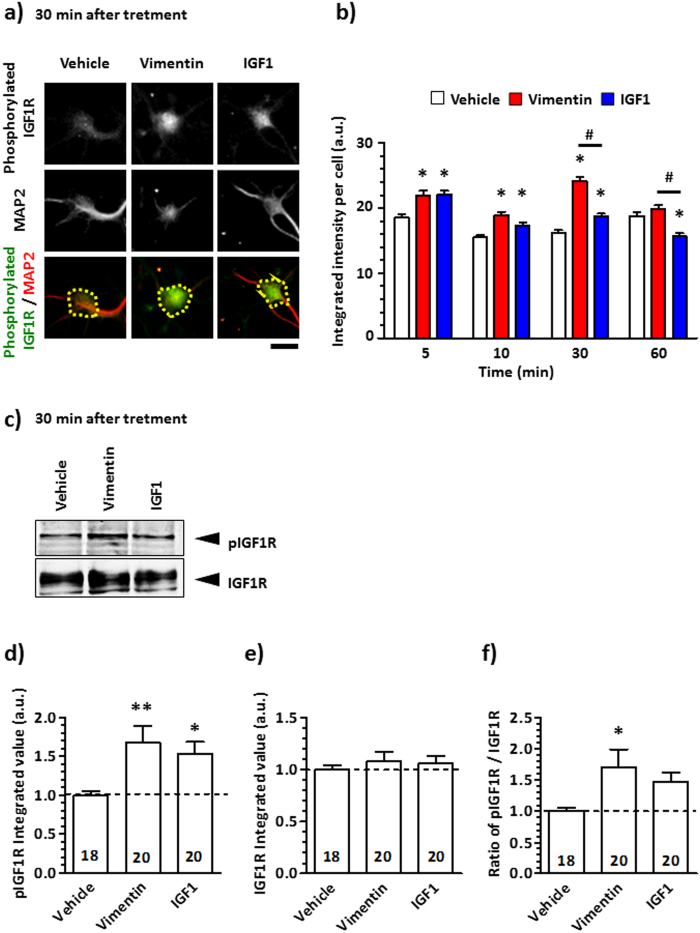
Tyrosine phosphorylation of the IGF1R is induced by vimentin. The primary cultured cortical neurons were cultured for 4 days and then treated with 100 ng/ml vimentin, 10 ng/ml IGF1 or vehicle solution for 5, 10, 30 and 60 min. (**a**) Representative images show that the cells were immunostained for phosphorylated IGF1R (green) and MAP2 (red) after 30 min treatment. (**b**) Signal intensities of MAP2-positive phosphorylated IGF1R were quantified in the yellow dotted area at each time point. The scale bar indicates 20 μm (**a**). ^*^p < 0.05 vs. the vehicle-treated group, ^#^*p* < 0.05, two-way ANOVA with post hoc Bonferroni test. Analysed cell numbers in each treatment are shown as follows in the order of the 5, 10, 30 and 60 min treatments. Vehicle: 478, 482, 425 and 366; Vimentin: 322, 351, 484 and 469; IGF1: 482, 478, 566 and 397. (**c**) After 30 min treatment with 100 ng/ml vimentin or 10 ng/ml IGF1 or vehicle solution, the phosphorylated IGF1R and total IGF1R were detected by western blotting. (**d,e**) The band intensities of phosphorylated IGF1R and IGF1R were analysed. (**f**) The ratio of phosphorylated IGF1R to IGF1R was calculated. The number in each column indicates the number of analysed bands, ^*^p < 0.05 vs. the vehicle-treated group; ^**^p < 0.05, one-way ANOVA with post hoc Dunnett’s test.

We next investigated the time course of IGF1R phosphorylation by vimentin treatment. Mouse cortical neurons were cultured for 4 days and then treated with vimentin, IGF1 or vehicle solution for 5, 10, 30 and 60 min. After treatment, the cells were fixed and immunostained for phosphorylated IGF1R (Fig. 2a,b). Phosphorylated IGF1R levels in the vimentin-treated group were significantly higher than those in vehicle-treated group at 5, 10 and 30 min, and levels peaked at 30 min after treatment. The IGF1-induced IGF1R phosphorylation was significantly increased at 5, 10 and 30 min, and the levels were significantly lower than those induced by vimentin treatment at 30 and 60 min. At 30 min treatment, phosphorylated IGF1R and total IGF1R were detected by western blotting (Fig. 2c–f). The levels of phosphorylated IGF1R were significantly increased in vimentin and IGF1 treated groups (Fig. 2c,d). In contrast, the levels of total IGF1R were not changed by either treatment (Fig. 2c,e). The ratio of phosphorylated IGF1R to total IGF1R was significantly increased by vimentin treatment (Fig. 2f). These results suggest that vimentin interacts with IGF1R, and may lead to IGF1R autophosphorylation.

### Vimentin does not enhance IGF1, IGF2 or insulin secretion

In addition to IGF1, IGF2 and insulin are physiological ligands for IGF1R with different affinity^9^. To investigate the possibility that vimentin-induced IGF1R phosphorylation was mediated by intrinsic IGF1, IGF2 or insulin secretion from cultured neurons, the concentration of each factor in the conditioned medium was measured at 5, 10 or 30 min after vimentin treatment because vimentin increased IGF1R phosphorylation at those time points after vimentin treatment. No IGF1 or IGF2 was detected in vimentin-treated media at any time point in an enzyme-linked immunosorbent assay (ELISA) (Table 1 and Table 2), indicating that the IGF1 or IGF2 concentration in the media was below 3.5 pg/ml or 1.5 pg/ml, respectively (the detection limit in the ELISA kit). Insulin is contained in the B-27 supplement^10^, thus, each medium sample was diluted and then analysed. The insulin concentration was not increased by vimentin treatment (Table 3). These results suggest that IGF1R activation by vimentin was not mediated by secreted IGF1, IGF2 or insulin.

### Vimentin binds to IGF1R directly

**Figure 3:**
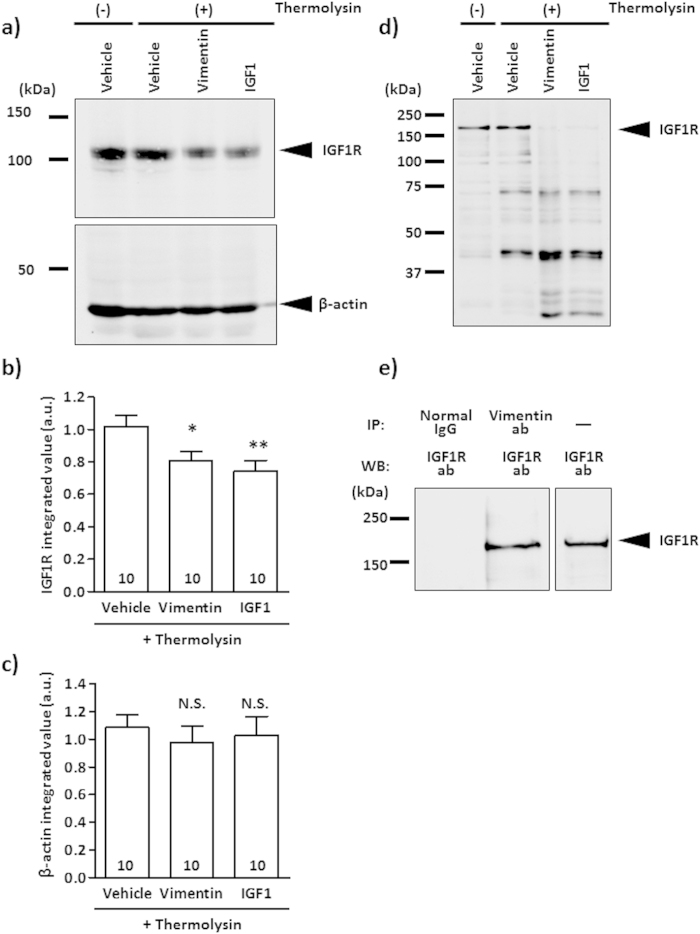
Vimentin binds to IGF1R directly. (**a–c**) Mouse cerebral cortex (ddY, adult) lysates were treated with vimentin, IGF1 or vehicle solution and incubated for 60 min at room temperature. Then, the mixture was treated with thermolysin and run on an SDS-PAGE gel. IGF1R was detected by western blotting (**a**). Bands of 95 kDa were detected as IGF1R. As a negative control, β-actin (45 kDa) was measured by western blotting. Band intensities of IGF1R (**b**) and β-actin (**c**) in vimentin-treated and IGF1-treated lysates were compared with that of vehicle solution-treated lysate under thermolysin-reacted conditions. The values were calculated relative to the absence of the thermolysin group. The numbers in the columns indicate the number of experiments independently performed. (**d**) Recombinant IGF1R protein was treated with vimentin, IGF1 or vehicle solution and incubated for 60 min at room temperature. After treatment, the mixture was reacted with thermolysin and run on an SDS-PAGE gel. IGF1R was detected by western blotting. Bands of approximately 150 kDa were detected as IGF1R. (**e**) Co-incubated recombinant IGF1R (5 pmol) and vimentin (10 pmol) w**e**re immunoprecipitated with anti-vimentin antibody or normal mouse IgG, and then IGF1R was detected by western blotting. As a control, only recombinant IGF1R (0.1 pmol) without IP was loaded on the separated right lane. ^*^p < 0.05 or ^**^p < 0.01 vs. thermolysin-treated vehicle group with a one-way ANOVA, *post hoc* Dunnett’s test. N.S. indicates not significant.

We performed a drug affinity responsive target stability (DARTS) analysis to investigate the direct binding of vimentin to IGF1R. If a ligand binds to a target protein, the stability of the target protein against protease is changed^11^, exhibiting thinner or thicker protein bands in western blot analysis^11^,^12^,^13^,^14^. Cerebral cortical lysates were incubated with vimentin, IGF1 or vehicle solution. Then, thermolysin (a protease) was added to the lysates. After the proteolysis reaction, the lysates were run on sodium dodecyl sulphate polyacrylamide gel electrophoresis (SDS-PAGE), and IGF1R levels were detected by western blotting (Fig. 3a). After thermolysin treatment, the 95-kDa IGF1R bands were obviously thinner in vimentin-treated and IGF1-treated lysates compared with the vehicle solution-treated lysates (Fig. 3a, upper panel). At that time, the β-actin levels were also checked as an internal control (Fig. 3a, lower panel). The band intensities of IGF1R and β-actin were quantified (Fig. 3b,c). The IGF1R levels in vimentin-treated and IGF1-treated lysates were significantly decreased compared with the vehicle solution-treated group (Fig. 3b). In contrast, the β-actin levels in the thermolysin-treated condition were similar among groups (Fig. 3c). A similar experiment was also performed using recombinant IGF1R protein instead of cerebral cortical lysates. After thermolysin treatment, the approximately 150-kDa recombinant IGF1R bands were notably thinner in the vimentin-treated and IGF1-treated groups compared with the vehicle solution-treated group (Fig. 3d). These results indicate that the direct interaction of vimentin with IGF1R changed the susceptibility of IGF1R to thermolysin, as did IGF1 treatment.

In addition, we confirmed the binding of vimentin to IGF1R with an immunoprecipitation analysis. Following the co-incubation of recombinant vimentin and recombinant IGF1R proteins, the samples were treated with anti-vimentin antibody or normal mouse IgG-conjugated protein G. The precipitated proteins were loaded on SDS-PAGE, after which IGF1R was detected by western blotting (Fig. 3e). By immunoprecipitation with anti-vimentin antibody, IGF1R was clearly co-precipitated, but not with normal mouse IgG. Only recombinant IGF1R was loaded as a control in a separate right lane. Thus, these results indicate that vimentin and IGF1R form a complex.

### Molecular dynamics (MD) simulations show that 330-407 residues of vimentin are presumed binding sites to IGF1R

To estimate the binding property of vimentin to IGF1R, an MD simulation was performed. First, the complex structure of IGF1R with IGF1 was constructed by referring to the known X-ray 3D structure of insulin and the insulin receptor. The binding free energy for the complex of the IGF1 molecule and IGF1R was −12.9736 kcal/mol. Because vimentin (466 amino acids) has 4 α-helix structures, those four parts of vimentin (99-189: PDB ID 3s4rB, 146-249: PDB ID 3uf1A, 261-335: PDB ID 3trtA and 330-407: PDB ID 1gk4D) were used for an MD simulation as a complex with IGF1R.

As shown in Table 4, the C-terminal portion of vimentin (330–407 amino acids) showed the lowest binding free energy (−9.9441), suggesting that this site is the most appropriate binding domain for IGF1R.

**Figure 4:**
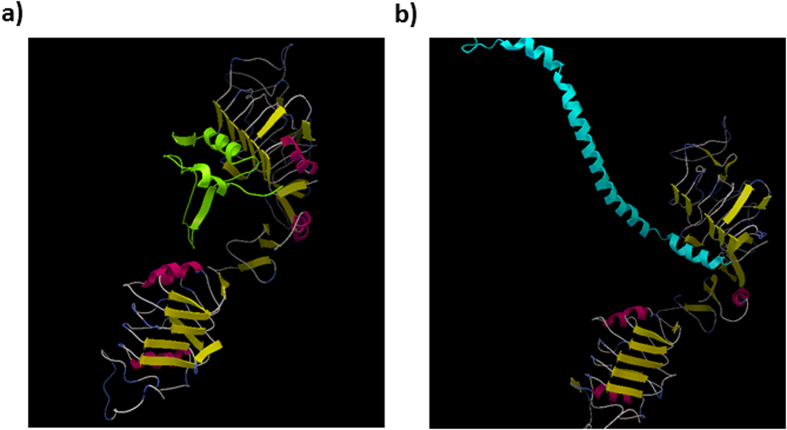
Supposed binding complex of IGF1 and vimentin with IGF1R. The complex of (**a**) IGF1 molecule (green) or (**b**) C-terminal (330–407 amino acids) of vimentin (cyan) with IGF1R (yellow and pink) was shown by MD simulation.

Vimentin has an extended rod-like structure consisting of repeated α-helical coils. The representative complex of IGF1 and vimentin with IGF1R is shown in Fig. 4.

## Discussion

We previously demonstrated that extracellular vimentin was secreted from astrocytes and promoted axonal growth^6^. Here, we showed that extracellular vimentin binds to IGF1R directly and facilitates axonal growth in cultured cortical neurons. This is the first report of the mechanism of action of extracellular vimentin in neurons.

Many groups have reported that the IGF1-IGF1R signalling pathway is important for enhancement of neurite outgrowth^15^,^16^,^17^,^18^,^19^,^20^,^21^. To activate this signalling pathway, the autophosphorylation of Tyr1135 and Tyr1136 in the β-subunit of IGF1R is essential because it mediates the stabilisation of the activated loop, leading to a conformational change of IGF1R^22^,^23^. In addition, this IGF1R activation is a critical step to inducing neurite outgrowth by IGF1^18^,^24^,^25^. Figure 2 shows that vimentin or IGF1 treatment significantly increased the phosphorylation levels of IGF1R at Tyr1135 and Tyr1136. Figure 1 shows that vimentin and IGF1 elicited similar levels of axonal growth at similar doses (1.75 nM, 1.3 nM, respectively), and these effects were diminished by two types of specific IGF1R inhibitors^26^: an IGF1R-neutralising antibody^7^ and an IGF1 analogue^8^,^27^. IGF1R-mediated axonal outgrowth has been reported to occur via the PI3-kinase/Akt signalling pathway^18^,^19^ or the MAPK signalling pathway^25^. Considering that extracellular vimentin increased IGF1R phosphorylation, vimentin appeared to induce axonal growth via a signalling pathway similar to IGF1.

Several groups have reported that DARTS analysis reveals alterations in the proteolytic responsiveness of receptors after ligand-receptor binding^11^,^12^,^13^,^14^. When insulin and insulin receptor (IR) bind, the IR is degraded by protease more readily than prior to insulin binding^28^. A comparison of amino acid sequences between IR and IGF1R demonstrated an 84% homology in their tyrosine kinase domains and a 44% homology in their carboxy-terminal domains^29^,^30^,^31^. The IGF1-IGF1R-bound form is assumed to be similar to the insulin-IR-bound form^32^. These data suggest that IGF1R is easily digested by a protease after binding to its ligands, IGF1 and vimentin, as shown in Fig. 3a,b. We used the full-length recombinant IGF1R, which had 1367 amino acids in DARTS analysis, as shown in Fig. 3d. IGF1R is synthesised as a pro-protein, and a 30-amino acid peptide of 1367 amino acids is cleaved after translation^33^. It is unknown whether the pro-protein of 30 amino acids and the mature IGF1R have different binding affinities to a ligand. However, our results showed that vimentin directly influenced the sensitivity of IGF1R to proteases. Furthermore, immunoprecipitation using an anti-vimentin antibody supports the hypothesis that vimentin and IGF1R form a complex (Fig. 3e).

Although the amino acid sequence of vimentin does not have any homology with IGF1, MD simulations also support that vimentin directly binds to IGF1R as IGF1 does. The representative structure of the IGF1R-IGF1 complex was superimposed onto the representative structures of the IGF1R-vimentin complexes to compare their spatial similarity. The root mean square deviations (RMSDs) of Cα atomic coordinates near IGF1R were 1.626 Å for residue number 99–189, 1.017 Å for residue number 146–249 and 1.161 Å for residue number 330–407 (the average score of the RMSDs was 1.268 Å). The RMSD for residue number 261–335 was not calculated because of its secondary structure breaking. The contact part of vimentin for residue number 330–407 is the C-terminus region (Fig. 4b). The accessibility of vimentin for residue number 330–407 to the contact area of IGF1R would be greater than other parts of vimentin because the terminal region of the protein is more flexible than other internal regions in general. We speculate that the contact region of vimentin to IGF1R may be its C-terminal region because of the lower RMSD for residue numbers 330–407, the lowest binding free energy and its accessibility to the contact area of IGF1R.

These results show that the IGF1R receptor is newly categorised as a “compatible receptor”. The three-dimensional partial structure of IGF1R has previously been demonstrated, and the approximate binding sites were identified^32^,^34^,^35^. Although the actual binding pocket and ligand-binding complex conformation had not been determined yet, our present data provided the assumed binding site of extracellular vimentin-IGF1R and the structure of the vimentin-binding IGF1R complex: This information should help with the analysis of the binding compatibility with IGF1R.

We previously indicated a role for extracellular vimentin *in vivo*^6^. In that study, we clarified that axons were elongated along with astrocytes that express and secrete vimentin in spinal cord-injured mice. Axonal growth in the injured spinal cord may be due to vimentin signalling via IGF1R. Furthermore, several reports have shown that IGF1 administration also promotes axonal growth in several nerve injury models^15^,^17^,^18^,^19^,^36^. After injury, IGF1 is primarily derived from activated microglia^37^. Vimentin and IGF1 are believed to be secreted from astrocytes and microglia, respectively, after which both proteins interact with IGF1R, leading to axonal growth. In the developmental stages, vimentin is widely expressed in the central nervous system^38^ and is believed to maintain the structural processes^1^. Several groups have reported that vimentin knockout mice showed no obvious phenotype^39^. Neurite length and neuronal viability were also not affected by vimentin knockout^40^ or knockdown^41^. Because the functional role of secreted vimentin was not considered in these studies using vimentin-deficient mice, the physiological contribution of extracellular vimentin in the developmental axonal guidance is unclear. However, other ligands for IGF1R such as IGF1, IGF2 and insulin, might compensate for the normal function of vimentin in the case of vimentin deficiency.

We previously reported that vimentin was secreted from astrocytes^6^. In that paper, the protein levels in astrocytes were compared between denosomin- (a novel compound) and vehicle-treated astrocytes using two-dimensional (2D)-PAGE and LC-MS/MS analyses. These analyses revealed that the levels of vimentin notably increased in denosomin-treated astrocytes. An ELISA confirmed the increased vimentin levels in a denosomin-treated astrocyte-conditioned medium. In this LC-MS/MS analysis, the phosphorylated peptide was not detected at all in vimentin-derived peptides. Thus, our previous data suggest that secreted vimentin is not phosphorylated in the extracellular space. However, Mor-Vakinin *et al.*^3^ showed that vimentin secretion is dependent upon phosphorylation in activated macrophages. Another group has also reported that vimentin is phosphorylated by PKCβ and then secreted from MCP-1 (monocyte chemotactic protein-1)-activated human monocytes^42^. However, neither reports investigated the possibility of vimentin secreted from astrocytes. Although several other groups reported that vimentin was secreted from astrocytes, no report indicated whether secreted vimentin was phosphorylated. Whether the secretion of vimentin from astrocytes is dependent upon its phosphorylation is the subject of further investigation.

Our findings indicate that extracellular vimentin, which is generally known as an intracellular protein, is a promising IGF1R ligand that promotes axonal growth as well as IGF1. These data provide novel knowledge regarding extracellular vimentin and a compatible role for IGF1R, suggesting a potential axonal growth strategy.

## Materials and Methods

All experiments were performed in accordance with the Guidelines for the Care and Use of Laboratory Animals at the Sugitani Campus of the University of Toyama and the NIH Guidelines for the Care and Use of Laboratory Animals. The Committee for Animal Care and Use at the Sugitani Campus of the University of Toyama approved the study protocols. All efforts were made to minimise the number of animals used.

### Primary cultures

Primary cultured cerebral cortical neuronal cells were prepared from E18 embryos of Sprague-Dawley rats (Japan SLC, Hamamatsu, Japan) (at a density of 2.55 × 10^5^ cells/cm^2^) on 100 × 20 mm tissue culture dishes (Falcon, Franklin Lakes, NJ, USA) or E14 embryos of ddY mice (Japan SLC) (at a density of 1.43 × 10^4^ cells/cm^2^) on 8-well chamber glass slides (Falcon) coated with 5 μg/ml poly-_D_-lysine (Sigma-Aldrich, St. Louis, MO, USA) as described previously^43^. Cortical neurons were cultured in neurobasal medium (Invitrogen, Carlsbad, CA, USA) containing 2% B-27 supplement (Invitrogen), 33 mM _D_-glucose (Wako, Osaka, Japan) and 2 mM _L_-glutamine (Wako) at 37 °C in a humidified incubator with 10% CO_2_.

### Measurement of axonal density

The cultured neurons at 1 day *in vitro* were treated with 10, 100 or 200 ng/ml vimentin (ProSpec, Rehovot, Israel) or 1, 5, 10 or 100 ng/ml IGF1 (Wako). In addition, the cells were treated with or without 2 μg/ml IGF1R-neutralising antibody (R&D systems, MS, USA) with or without 20 μg/ml IGF1 analogue (BACHEM, Bubendorf, Switzerland) for 15 min. Then, 100 μg/ml vimentin or 10 μg/ml IGF1 was added. After 6 days of treatment, the cells were fixed with 4% paraformaldehyde and immunostained with a mouse anti-phosphorylated neurofilament-H (pNF-H) monoclonal antibody (clone: SMI-35, dilution 1:500; Covance, Emeryville, CA, USA) as an axonal marker and a rabbit anti-microtubule-associated protein 2 (MAP2) polyclonal antibody (dilution 1:1000, Abcam, Cambridge, United Kingdom) as a neuronal marker. Alexa Fluor 594-conjugated goat anti-mouse IgG (dilution 1:400; Invitrogen) and Alexa Fluor 488-conjugated goat anti-rabbit IgG (dilution 1:400; Invitrogen) were used as secondary antibodies. Neurons were counterstained with 1 μg/ml DAPI (Enzo Life Sciences, Farmingdale, NY, USA). Fluorescent images were captured using a fluorescent microscopy system (BX-61/DP70, Olympus, Tokyo, Japan) at 640 μm × 850 μm. The lengths of the pNF-H-positive axons were measured using a Neurocyte image analyser (Kurabo, Osaka, Japan) that automatically traces pNF-H-positive axons and measures axon length in the image. The sum of the axon lengths was divided by the number of MAP2-positive neurons in the image. The axon densities were averaged over all the images and are shown in Fig. 1.

### Exploration of phosphorylated protein by vimentin

The Phospho Explorer Antibody Microarray kit (Full Moon BioSystems, Sunnyvale, CA, USA) was used to investigate phosphorylated proteins. Four days after the culture of rat cortical neurons, vimentin (100 ng/ml) or vehicle solution (distilled water) was administered to the neurons for 10 min. After washing with cold PBS, the extraction buffer in the kit was added to the cultured neurons and incubated for 1 h on ice with occasional mixing. The extracts were centrifuged at 17,968 × *g* for 20 min at 4 °C, and the supernatants were saved as cell lysates. Biotin-labelling reactions were performed for 2 h at room temperature and stopped by incubation with the kit stop regent for 30 min. The labelled protein solution was coupled on slides in the kit for 2 h at room temperature. Each slide was coated with a total of 656 phospho- and non-phospho-antibodies spotted in duplicate. Then, each slide was incubated in the detection buffer containing C3-streptavidin for 45 min in the dark. The fluorescent intensities of the proteins were quantified by the arrays on Axon GenePix scanners (Filgen, Nagoya, Japan).

### Detection of IGF1R phosphorylation

IGF1R phosphorylation was detected by immunocytochemical analysis and western blotting. Mouse cortical neurons (E14) were cultured in 8-well chamber slides or 6-cm dishes at a density of 1.43 × 10^4^ cells/cm^2^ or 3.53 × 10^5^ cells/cm^2^, respectively for 4 days. In the immunocytochemical analysis, vimentin (100 ng/ml), IGF1 (10 ng/ml) or vehicle solution (distilled water) was administered to the cells cultured in 8-well chamber slides for 5, 10, 30 and 60 min, and the cells were immunostained with a rabbit anti-IGF1R (phospho-Tyr1165/Tyr1166) antibody (0.005 μg/ml, Full Moon BioSystems) and a MAP2 monoclonal antibody (clone: HM-2, dilution 1:500, Merck Millipore, Billerica, MA, USA) as a neuronal marker. Alexa Fluor 594-conjugated goat-anti mouse IgG (1:400) and Alexa Fluor 488-conjugated goat-anti-rabbit IgG (1:400) were used as secondary antibodies. Neurons were counterstained with 1 μg/ml DAPI. Images were captured using a fluorescence microscope system (BX-61/DP70) at 324 μm × 430 μm. IGF1R phosphorylation was quantified as the fluorescent intensity in the neuron cell body using MetaMorph 7.8 (Molecular Devices, Sunnyvale, CA, USA). In case of detection of IGF1R phosphorylation by western blotting, vimentin (100 ng/ml), IGF1 (10 ng/ml) or vehicle solution (distilled water) was administered to the cells cultured in 6-cm dishes for 30 min, and then the cells were washed once with cold PBS. Then, the cells were incubated with M-PER lysis buffer (Thermo Fisher Scientific, Waltham, MA, USA) containing a protease and phosphatase inhibitor cocktail (Thermo Fisher Scientific) for 30 min. After the incubation, cells were centrifuged (14,000 × *g*, 10 min, 4 °C), and supernatants were used as cell lysates. The samples were combined with NuPAGE LDS sample buffer (Life Technologies, Carlsbad, CA, USA) and 10% 2-mercaptoethanol and loaded on an 8% SDS/polyacrylamide gel. After electrophoresis, the proteins were transferred to a nitrocellulose membrane and blocked for 1 h at room temperature in 0.1% Tween 20-containing TBS (T-TBS) supplemented with 3% ECL prime blocking regent (Amersham, Amersham, UK). Subsequently, the membrane was briefly washed with T-TBS and incubated with rabbit anti-IGF1R (phospho-Tyr1165/Tyr1166) rabbit polyclonal antibody (Full Moon BioSystems) at a 1:1000 dilution in immunoreaction buffer (Can Get Signal solution 1, Toyobo, Osaka, Japan) overnight at 4 °C. After washing with T-TBS, the membrane was incubated with the secondary antibody (goat anti-rabbit IgG-HRP, Santa Cruz, Dallas, TX, USA) at a 1:2000 dilution in immunoreaction buffer (Can Get Signal solution 2, Toyobo) for 1 h at room temperature. Using the detection regent (ECL prime regent, GE Healthcare, Pittsburgh, PA, USA), the chemiluminescence signals on the membrane were detected in LAS4000 (GE Healthcare). Then, the antibodies were stripped from the membrane using western blotting stripping solution (Nacalai Tesque, Kyoto, Japan). After stripping, the membrane was blocked with 5% nonfat dry milk and incubated with the primary antibody against IGF1R (clone: D23H3, rabbit monoclonal IgG, Cell Signaling Technology, Danvers, USA) at a 1:1000 dilution in immunoreaction buffer (Can Get Signal solution 1) overnight at 4 °C. After washing with T-TBS, the membrane was incubated with the secondary antibody (goat anti-rabbit IgG-HRP, Santa Cruz) at a 1:2000 dilution in immunoreaction buffer (Can Get Signal solution 2) for 1 h at room temperature. Using the detection regent (ECL prime regent), the chemiluminescence signals on the membrane were detected in LAS4000. The signal intensities were quantified using a CS analyser (ATTO, Tokyo, Japan).

### Enzyme-linked immunosorbent assay (ELISA) for IGF-1, IGF2 or insulin in conditioned medium of vimentin-treated cultured cortical neurons

Mouse cortical neurons (ddY, E14) were cultured in 8-well chamber slides for 4 days. Vimentin (100 ng/ml) or vehicle solution (distilled water) was administered to the cells for 5, 10 or 30 min. The cultured medium was collected and centrifuged at 15,000 rpm for 10 min at 4 °C. The supernatant was collected as conditioned medium. The B27 supplement contains insulin, thus, each sample was diluted with distilled water at a 1:500 dilution. The IGF1, IGF2 or insulin concentrations in the conditioned medium were quantified using a mouse IGF1 ELISA kit (R&D Systems, Minneapolis, MN, USA), mouse IGF2 ELISA kit (R&D Systems) or mouse insulin ELISA kit (Mercodia, Uppsala, Sweden), respectively according to the manufacturers’ protocols.

### Drug affinity responsive target stability (DARTS) experiments

Mice (ddY, 8–12 weeks old, male and female) were anaesthetised and perfused with cold physiological saline. The cortices were removed from the skull and homogenised with M-PER lysis buffer (Thermo Fisher Scientific). After centrifugation (14,000 × *g*, 10 min, 4 °C), the supernatants were mixed with vimentin, IGF1 or vehicle solution (lysate:drug, 100 μg:1 pmol) and incubated for 60 min at room temperature. Each sample was proteolysed with thermolysin (Sigma-Aldrich) in reaction buffer [50 mM Tris▪HCl (pH 8.0), 50 mM NaCl, 10 mM CaCl_2_] for 10 min at 37 °C (thermolysin:sample, 1 μg:10^5^ μg). To halt the proteolysis reaction, 0.5 M ethylene diaminetetraacetic acid (EDTA) (pH 8.0) was added to each sample in one-tenth volume. Then, the samples were concentrated by centrifugal filter devices (Amicon Ultra 10K devices, Merck Millipore, Darmstadt, Germany).

Recombinant human IGF1R protein in 25 mM Tris-HCl (pH 8.0) containing 2% glycerol (full length, Abnova, Taipei, Taiwan) was mixed with vimentin, IGF1 or vehicle solution (IGF1R protein:drug, 1 ng:19 ng) and incubated for 60 min at room temperature. Each sample was proteolysed with thermolysin (Sigma-Aldrich) in the reaction buffer for 10 min at 37 °C (thermolysin:sample, 1 ng:50 ng). To halt the proteolysis reaction, 0.5 M EDTA (pH 8.0) was added to each sample in one-tenth volume.

After the reaction was stopped, the reaction solutions were reacted with 4 × sample buffer at 95 °C for 5 min and then loaded onto SDS polyacrylamide gel (8% separating gel). After electrophoresis, the proteins were transferred to a nitrocellulose membrane and blocked for 1 h at room temperature in 0.1% Tween 20-containing TBS (T-TBS) supplemented with 5% nonfat dry milk. Subsequently, the membrane was briefly washed with T-TBS and incubated with the primary antibody against IGF1R (clone: D23H3, rabbit monoclonal IgG, Cell Signaling Technology) at a 1:1000 dilution in immunoreaction buffer (Can Get Signal solution 1) overnight at 4 °C. After washing with T-TBS, the membrane was incubated with the secondary antibody (goat anti-rabbit IgG-HRP, Santa Cruz) at a 1:2000 dilution in immunoreaction buffer (Can Get Signal solution 2) for 1 h at room temperature. Using the detection regent (ECL prime regent), the chemiluminescence signals on the membrane were detected in LAS4000. Signal intensities were quantified using a CS analyser (ATTO).

### Binding experiment of vimentin and IGF1R by immunoprecipitation

Recombinant vimentin protein (10 pmol) and recombinant IGF1R protein (5 pmol) were co-incubated for 1 h at room temperature with rotation. Fifty microliters of Dynabeads Protein G (Life technologies) was treated with 1% bovine serum albumin (BSA) for blocking in M-PER protein extraction regent solution (Thermo Fisher Scientific) for 2 h at room temperature with rotation. Then, the protein G was incubated with anti-vimentin antibody (clone: RV202, 1 μg, mouse monoclonal IgG, Santa Cruz) or normal mouse IgG (Santa Cruz) for 1 h at room temperature with rotation. After the protein G was washed with M-PER solution, incubated vimentin and IGF1R were added to the washed protein G followed by continuous rotation over night at 4 °C. Each sample was eluted by NuPAGE LDS sample buffer and was separated on 8% SDS/polyacrylamide gel and transferred to a nitrocellulose membrane. The membrane was blocked by 5% nonfat dry milk (Wako, Tokyo, Japan). Subsequently, the membrane was briefly washed with T-TBS and incubated with the primary antibody against IGF1R (clone: D23H3, rabbit monoclonal IgG, Cell Signaling Technology) at a 1:1000 dilution in immunoreaction buffer (Can Get Signal solution 1) overnight at 4 °C. After washing with T-TBS, the membrane was incubated with the secondary antibody (goat anti-rabbit IgG-HRP, Santa Cruz) at a 1:2000 dilution in immunoreaction buffer (Can Get Signal solution 2) for 1 h at room temperature with rotation. For the anti-rabbit IgG-HRP antibody, bands were visualised with Amersham ECL detection regent (GE Healthcare) and detected by LAS4000 (GE Healthcare).

### Molecular dynamics (MD) simulations and binding free energy calculation

Protein Databank (PDB) data were used as the initial structure data for IGF1R (PDB ID: 1IGR) and IGF1 (PDB ID: 1GZY). The missing data in the proteins were restored using the homology modelling method in MODELLER^44^. The complex structure of IGF1R with IGF1 was constructed by superimposing and rearranging it with the complex structure of the insulin receptor with insulin (PDB ID: 3W14) as a template using web server MATRAS^45^. Four types of PDB data (PDB ID: 3s4rB, 3uf1A, 3trtA and 1gk4D) that are matched to the regions of vimentin (99–189, 146–249, 261–335 and 330–407, respectively) were used as initial structures of vimentin. The complex structures of IGF1R with the four parts of vimentin were constructed as above.

The complex structures were used as starting structures for the MD simulation by Amber12^46^ with an ff99SB force field. These structures were solvated in a cubic box of TIP3P water extending at least 10 Å in each direction from the solute, and the cut-off distance was maintained at 12 Å to compute the nonbonded interactions. All of the complex structures were performed under periodic boundary conditions^47^, and the long-range electrostatic was treated by using the particle-mesh-Ewald method^48^,^49^. The time step was set to 1 fs, and the trajectory was recorded every 0.1 ps.

Prior to MD simulations, the systems were relaxed by a series of steepest descent (SD) and conjugated gradient (CG) minimisations. The 3-ns MD simulations were performed based on each of the minimised systems by gradually heating over 60 ps from 0 to 300 K with the protein atoms fixed using a force constant of 5 kcal/mol/Å2. Then, a 200-ps pressure-constant period (NPT) was applied to obtain an equilibrated density of the constrained protein atoms. The following step was a 40-ps-volume-constant period (NVT) at a force constant of 2.5 kcal/mol/Å2 followed by 100 ps dynamics at a force constant of 1.25 kcal/mol/Å2. Finally, a 1.6-ns unrestrained MD simulation (no force applied on any protein atoms) was performed for each fully flexible system in the NVT ensemble at a constant temperature of 300 K. A total of 500 snapshots were collected at 1-ps-intervals from the last 500 ps of MD for the binding free energy analysis.

Based on the selected MD snapshots, the binding free energies for each complex were estimated using MM-GBSA (Molecular Mechanics Generalized Born Solvent Area)^50^. The binding free energies (ΔG_binding_) were determined from the free energies of the complex, protein and peptide according to Equation (1):
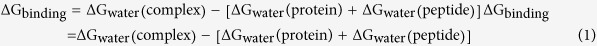


### Statistical analyses

All results are expressed relative to the control value. All experiments were performed at least two to three times. The results are shown as the mean ± S.E. The statistical comparisons between groups were analysed by one-way ANOVA (Figs 1a–f, 2d–f and 3b,c) or two-way ANOVA (Fig. 2c) using GraphPad Prism 5.0 (GraphPad Software, La Jolla, CA, USA), and *p* values less than 0.05 were considered significant.

## Additional Information

**How to cite this article**: Shigyo, M. *et al.* Extracellular vimentin interacts with insulin-like growth factor 1 receptor to promote axonal growth. *Sci. Rep.* **5**, 12055; doi: 10.1038/srep12055 (2015).

## Supplementary Material

Supplementary Information

## Figures and Tables

**Table 1:** No IGF1 secretion occurred in vimentin-treated cortical neurons.

Treatment	Time after treatment	O.D.		IGF1 (pg/ml)	
		Mean	S.E.M.	Mean	S.E.M.
Blank	–	0.04618	0.00116		
Medium	–	0.04944	0.00311	N.D.	N.D.
Vehicle	5 min	0.04626	0.00269	N.D.	N.D.
	10 min	0.04394	0.00116	N.D.	N.D.
	30 min	0.04518	0.00141	N.D.	N.D.
Vimentin (100 ng/ml)	5 min	0.04620	0.00189	N.D.	N.D.
	10 min	0.04456	0.00114	N.D.	N.D.
	30 min	0.04164	0.00088	N.D.	N.D.

IGF1 concentrations in the conditioned medium were measured by ELISA. IGF1 concentrations in media were calculated using a standard curve (n = 5 per group). N.D. indicates not detectable.

**Table 2:** No IGF2 secretion occurred in vimentin-treated cortical neurons.

Treatment	Time after treatment	**O.D.**		**IGF2 (ng/ml)**	
		**Mean**	**S.E.**	**Mean**	**S.E.**
Blank	–	0.10358	0.00077		
Medium	–	−0.00191	0.00112	N.D.	N.D.
Vehicle	5 min	0.00742	0.00049	N.D.	N.D.
	10 min	0.00532	0.00423	N.D.	N.D.
	30 min	0.00059	0.00063	N.D.	N.D.
Vimentin (100 ng/ml)	5 min	0.01042	0.00066	N.D.	N.D.
	10 min	0.00322	0.00131	N.D.	N.D.
	30 min	0.00477	0.00380	N.D.	N.D.

IGF2 concentrations in the conditioned medium were measured by ELISA. IGF2 concentrations in media were calculated using a standard curve (n = 4 per group). N.D. indicates not detectable.

**Table 3:** The insulin concentration was not increased in culture medium by vimentin treatment.

Treatment	Time after treatment	**O.D.**		**Insulin (ng/ml)**	
		**Mean**	**S.E.**	**Mean**	**S.E.**
Blank	–	0.0698	0.0731		
Medium	–	1.0912	0.0731	2144.2470	125.2882
Vehicle	5 min	0.9452	0.1035	1885.0002	182.2244
	10 min	1.0771	0.1128	2113.1821	193.1973
	30 min	1.1008	0.1324	2148.6437	215.7230
Vimentin (100 ng/ml)	5 min	1.0611	0.1248	2083.0581	212.3034
	10 min	0.8247	0.0506	1672.9189	93.7380
	30 min	0.8824	0.0389	1780.3009	71.3818

Insulin concentrations in media were calculated using a standard curve (n = 4 per group). The insulin concentration in each treatment was not increased by vimentin treatment.

**Table 4:** **Calculated binding free energy.**

Ligand	**amino acids**	**PDB ID**	**Binding Free Energy (kdal/mol)**
IGF1	1–70	1GZY	−12.9736
Vimentin	99–189	3s4rB	−0.9959
Vimentin	146–249	3uf1A	2.7303
Vimentin	261–335	3trtA	2.8353
Vimentin	330–407	1gk4D	−9.9441

The binding free energy for each complex was estimated using MM-GBSA.
