# Supplementary material for: Extracellular vimentin interacts with insulin-like growth factor 1 receptor to promote axonal growth
Source: Sci Rep. 2015 Jul 14;5:12055. doi: 10.1038/srep12055 (PMC4501001; doi:10.1038/srep12055)
Supplement: Supplementary Information [file srep12055-s1.pdf]

Supplementary Information

Title: Extracellular vimentin interacts with insulin-like growth factor 1 receptor to promote axonal growth

Michiko Shigyo[1], Tomoharu Kuboyama[1], Yusuke Sawai[2], Masahito Tada-Umezaki[2] & Chihiro Tohda[1*]

[1]Division of Neuromedical Sciences, Institute of Natural Medicine, University of Toyama, 2630 Sugitani, Toyama 930-0194, Japan

[2]Division of International Cooperative Research, Research Center for Ethnomedicine, Institute of Natural Medicine, University of Toyama, 2630, Sugitani, Toyama 930-0194, Japan

*Corresponding author: Chihiro Tohda, Ph.D.

Division of Neuromedical Sciences, Institute of Natural Medicine, University of Toyama, 2630 Sugitani, Toyama 930-0194, Japan

Tel: +81-76-434-7646

Fax: +81-76-434-7646

E-mail: chihiro@inm.u-toyama.ac.jp

E-mail address of the other authors

Michiko Shigyo: d1361304@ems.u-toyama.ac.jp

Tomoharu Kuboyama: kuboyama@inm.u-toyama.ac.jp

Yusuke Sawai: ysawa368@inm.u-toyama.ac.jp

Masahito Tada-Umezaki: masume@inm.u-toyama.ac.jp

Supplemental Methods

Measurement of neuronal cell density

The cultured neurons at 1 day *in vitro* were treated with 10, 100 or 200 ng/ml vimentin (ProSpec, Rehovot, Israel) or 1, 5, 10 or 100 ng/ml IGF1 (Wako). In addition, the cells were treated with or without 2 μg/ml IGF1R-neutralising antibody (R&D Systemsor, MS, USA) with or without 20 μg/ml IGF1 analogue (BACHEM, Bubendorf, Switzerland) for 15 min. Then, 100 μg/ml vimentin or 10 μg/ml IGF1 was added. After 6 days of treatment, the cells were fixed with 4% paraformaldehyde and immunostained with a mouse anti-phosphorylated neurofilament-H (pNF-H) monoclonal antibody (clone: SMI-35, dilution 1:500; Covance, Emeryville, CA, USA) as an axonal marker and a rabbit anti-microtubule associated protein 2 (MAP2) polyclonal antibody (dilution 1:1000, Abcam, Cambridge, United Kingdom) as a neuronal marker. Alexa Fluor 594-conjugated goat anti-mouse IgG (dilution 1:200; Invitrogen) and Alexa Fluor 488-conjugated goat anti-rabbit IgG (dilution 1:200; Invitrogen) were used as secondary antibodies. Neurons were counterstained with DAPI (Enzo Life Sciences, Farmingdale, NY, USA). Fluorescent images were captured using a fluorescent microscopy system (BX-61/DP70, Olympus, Tokyo, Japan) at 640 μm×850 μm. MAP2-positive neuronal cells in each photo were counted by using imageJ software.

Cell viability Assay

Cerebral cortical neurons were isolated from E14 mouse embryos and plated in poly-D-lysine coated 96-well plate at 2 x 10^4 cells/well in the Neurobasal medium (Invitrogen) contained 12% horse serum, 0.6 % D-glucose and 2 mM L-glutamine for 5 hs, then the medium was changed to Neurobasal medium containing 2 % B-27 supplement, 0.6 % D-

glucose and 2 mM L-glutamine. At four days *in vitro*, media was replaced with the Neurobasal medium containing vimentin (1 to 200 ng/ml) or IGF1 (1 to 100 ng/ml). By using the CellTiter-Glo® (Promega), luminescent levels of survival cells were measured.

Results

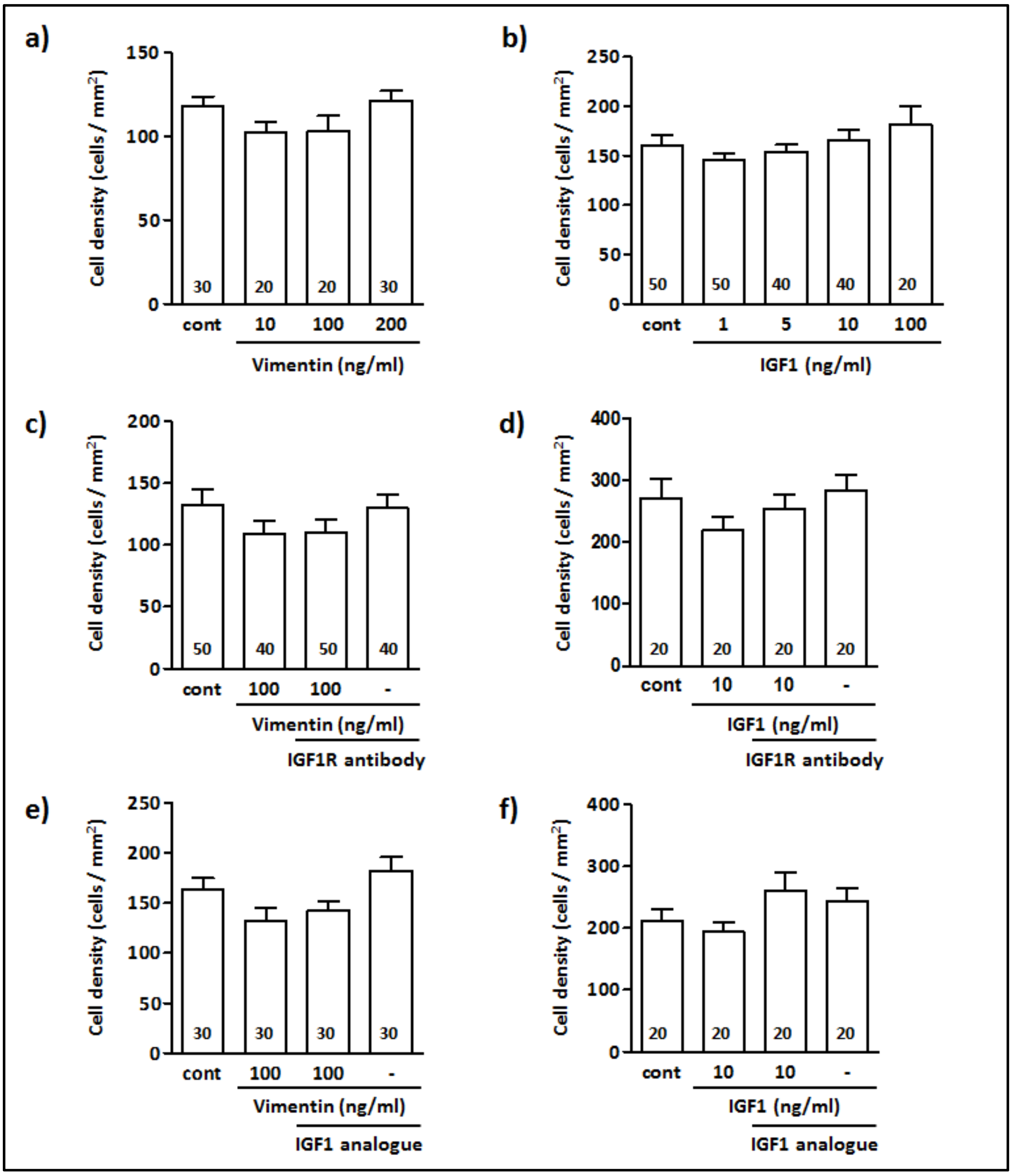

Supplementary Figure S1

Primary cultured cortical neurons at 1 day *in vitro*, vimentin (10, 100, 200 ng/ml) or IGF1 (1, 5, 10, 100 ng/ml) was treated following 15 minutes pretreatment with IGF1R neutralizing antibody (2 μg/ml) or IGF1 analogue (20 μg/ml). At 6 days after treatment, cells were fixed and immunostained with MAP2, and counter stained with DAPI. The number of column indicates number of photos.

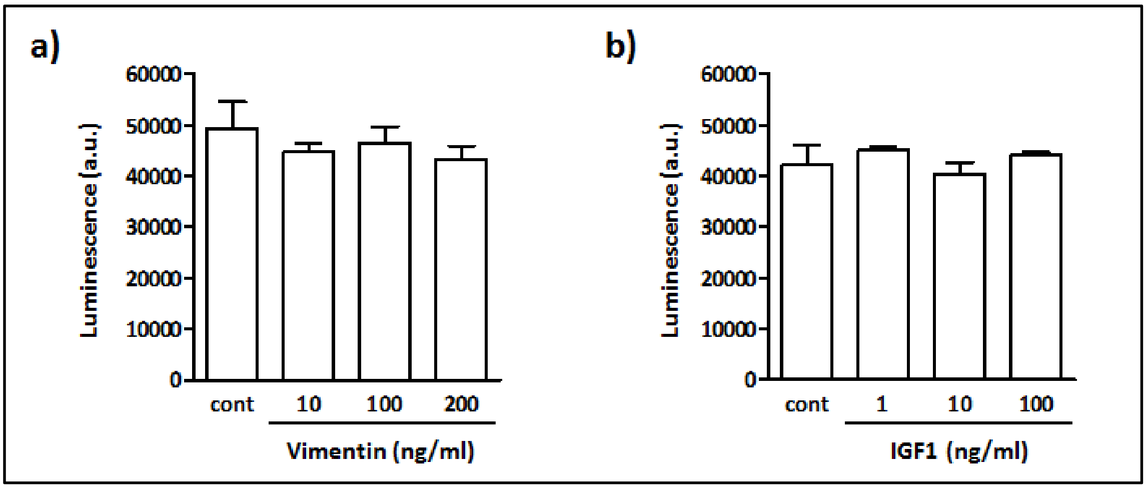

Supplementary Figure S2

Primary cultured cortical neurons at 4 days *in vitro*, vimentin (10, 100, 200 ng/ml) or IGF1 (1, 10, 100 ng/ml) was treated for 24 h, and then the luminescent signals of survival cells were detected by using CellTiter-Glo® regent. At 5 days *in vitro*, the number of neuronal cells was not significantly changed between groups.
